# Controlling of Mycobacterium by Natural Degradant-Combination Models for Sequestering Mycolic Acids in Karish Cheese

**DOI:** 10.3390/molecules27248946

**Published:** 2022-12-15

**Authors:** Gamal Hamad, Marwa A. Saad, Dalia Talat, Sabria Hassan, Ola M. A. K. Shalabi, Abeer M. Salama, Sarah Abou-Alella, Tuba Esatbeyoglu, Taha Mehany

**Affiliations:** 1 Department of Food Technology, Arid Lands Cultivation Research Institute, City of Scientific Research and Technological Applications, New Borg El-Arab 21934, Egypt; 2 Department of Food Hygiene and Control, Faculty of Veterinary Medicine, Menofia University, Shibeen El-Kom 32511, Egypt; 3 Department of Microbiology, Faculty of Veterinary Medicine, Damanhur University, Damanhur 22511, Egypt; 4 Dairy Department, Faculty of Agriculture, Mansoura University, Mansoura 33516, Egypt; 5 Department of Environmental Sciences, Faculty of Science, Port Said University, Port Said 42521, Egypt; 6 Department of Food Hygiene and Control, Faculty of Veterinary Medicine, Alexandria University, Alexandria 21523, Egypt; 7 Department of Food Development and Food Quality, Institute of Food Science and Human Nutrition, Gottfried Wilhelm Leibniz University Hannover, Am Kleinen Felde 30, 30167 Hannover, Germany

**Keywords:** *Mycobacterium tuberculosis*, karish cheese, probiotics, natural extract, fruit, functional ingredient, degradation, organic acid

## Abstract

Degradation of the *mycobacterial* complex containing mycolic acids (MAs) by natural bioactive compounds is essential for producing safe and value-added foods with therapeutic activities. This study aimed to determine the degradation efficiency of natural organic acid extracts (i.e., citric, malic, tartaric, and lactic), quadri-mix extract from fruits and probiotics (i.e., lemon, apple, grape, and cell-free supernatant of *Lactobacillus acidophilus*), and synthetic pure organic acids (i.e., citric, malic, tartaric, and lactic), against MA in vitro in phosphate buffer solution (PBS) and Karish cheese models. The degradation effect was evaluated both individually and in combinations at different concentrations of degradants (1, 1.5, and 2%) and at various time intervals (0, 6, 12, 24, and 48 h). The results show that MA degradation percentage recorded its highest value at 2% of mixed fruit extract quadri-mix with *L. acidophilus* and reached 99.2% after 48 h both in PBS and Karish cheese, unlike other treatments (i.e., citric + malic + tartaric + lactic), individual acids, and sole extracts at all concentrations. Conversely, organic acid quadri-mix revealed the greatest MA degradation% of 95.9, 96.8, and 97.3% at 1, 1.5, and 2%, respectively, after 48 h. Citric acid was more effective in MA degradation than other acids. The fruit extract quadri-mix combined with *L. acidophilus*-fortified Karish cheese showed the highest sensorial characteristics; hence, it can be considered a novel food-grade degradant for MA and could be a promising biocontrol candidate against *Mycobacterium tuberculosis* (Mtb) in food matrices.

## 1. Introduction

Tuberculosis (TB) is a global contagious infection, with an estimated 10 million new cases and 1.2 million deaths in 2019 [1]. It is induced by Mtb, a bacterium that causes several fatal cases, particularly in immunocompromised individuals [2]. Apart from humans, bovine TB is prevalent in dairy herds. Diseased animals can secrete milk containing *Mycobacterium bovis*, which is of great concern due to the potential ingestion of bacteria via milk and its products, which could lead to bovine TB in humans [3].

Several attempts to treat TB have failed due to the development of drug-resistant species of *Mycobacterium* spp. to anti-tubercular drugs. The hydrophobic components of the cell wall, such as MA, are one of the causes of mycobacterial resistance. Mycolic acid is the primary and distinctive lipidemic component of the mycobacterial cell membrane and is crucial for the viability, pathogenicity, and resistance of *Mycobacterium* spp. [4]. MA, two-alkyl, three-hydroxy long-chain fatty acid, is found either unbound or linked with the polysaccharide (arabinogalactan, AG) and peptidoglycan (PG) to create a robust cell wall frame, also known as the MA–AG–PG (mycolic acid–arabinogalactan–peptidoglycan) complex or the myco-membrane [5]. This complex is resistant to common antibiotics and can be attacked by agents with a high affinity for lipid-rich cell surfaces [6].

Utilizing hydrophobic biologically active compounds, such as organic acids, is effective in defeating the hydrophobic complex. Hence, the present research focuses on identifying organic acids with new anti-mycobacterial activity for improved efficacy, safety, and potency in the food industry. The FDA has categorized organic acids as “generally recognized as safe” (GRAS) to the public [7]. Organic acids are classified into monocarboxylic, dicarboxylic, alpha-hydroxyl, and sugar acids. In addition, they can have a variety of beneficial effects, including antioxidant, antimicrobial, anti-inflammatory, immunomodulation, and biodegradation activities [8].

Among organic acids (i.e., citric, malic, and tartaric acids), non-aromatic, short-chain alpha hydroxyl acids, e.g., lactic acid and glycolic acid, are primarily created in the Krebs cycle are essential for fruit physiology [9]. Numerous fruity matrices from Rutaceae, Rosaceae, and Vitaceae families, including lemon (*Citrus limon*), apples (*Malus communis*, *M. pumila*, and *M. sylvestris*), and grapes (*Vitis vinifera*, *V. labrusca*, and *V. rotundifolia*) contain a significant ratio of these acids [10]. Lactic acid is produced by lactic acid bacteria (LAB), primarily by *Lactobacillus* spp., during carbohydrate fermentation of foods such as dairy products and pickles, accounting for roughly 85% of its production associated with food production [11]. Organic acids are widely used as additives, preservatives, acidulates, flavor enhancers, emulsifiers, sequestrates, fermentative, and buffering media in various areas of manufacturing such as food, drinks, therapeutic, and beautifying products [12].

Acidification is essential in the food industry since it affects many dairy products in terms of aroma, taste, appearance, and shelf life. Among acidified dairy products, Karish, an artisanal Egyptian cheese, is made from skimmed milk by acidification [13]. Few studies have been conducted recently to investigate the capability of *Mycobacterium* spp. to survive during food processing processes such as pasteurization and fermentation, with varied results [14,15]. However, the degradation efficiency of organic acids against MA in the acid-coagulated food model (Karish cheese) has received little attention. In addition, Karish is a low-fat cheese and can display unpleasant sensory properties, such as fragility, friability, and increased or decreased syneresis [16]. In this study, we hypothesized that fortifying Karish cheese with organic acid extracts of fruits and LAB would enhance the sensory features of fortified cheese.

Organic acid extracts from plants, fruits, and bacteria have been described as an eco-friendly and alternative source of anti-mycobacterial compounds, which could be studied for advanced therapeutic approaches in a few recent studies [17,18]. However, the response of mycobacterial cell wall components to organic acid degradation activities has received little attention, particularly the capability of organic acids to degrade MA in vitro. However, it may be beneficial to investigate these degradation approaches, which may be critical for identifying unique natural anti-mycobacterial products applied in the food industry.

This study has revealed the first in vitro response of MA to organic acids, potent antimicrobial agents, as an approach to the destruction or restoration of the mycobacterial cell wall. Therefore, to fill this gap, we investigated the degradation abilities of citric, lactic, malic, and tartaric acids against MA by using HPLC, which had not been previously reported, in order to discover new anti-mycolic acid products. Furthermore, the organic acid sources in the study were purchased from fruit extracts of lemon, apple, and grape and cell-free supernatant of probiotic *Lactobacillus acidophilus*. Moreover, to the best of our knowledge, no study has investigated the application of degradant-combination models for sequestering MA in food matrices. In the same regard, there is currently no fortified cheese with extract quadri-mix available on the market as an eco-friendly dairy product with anti-mycolic acid activity, and there is no literature data on works on such food formulation.

Consequently, the novelty of our work is to evaluate and compare the effectiveness of citric, lactic, malic, and tartaric acids as an MA degradant in three models, sole, acid quadri-mix, and extract quadri-mix, in PBS based on time, type, and concentration of used acids. Furthermore, the degradation activity of extract quadri-mix from fruits and probiotics (i.e., lemon, apple, grape, and cell-free supernatant of *L. acidophilus*) was studied using the acid coagulated (Karish cheese) model, and the effect on the sensory properties of novel extract quadri-mix-fortified cheese was evaluated as well.

## 2. Results and Discussion

### 2.1. Mycolic Acid Degradation Efficiency by Organic Acids in PBS

MA degradation efficiency using individual and mixed forms of organic acids (OAs) (i.e., citric acid, malic acid, tartaric acid, and lactic acid) at various concentrations (1, 1.5, and 2%) in PBS buffer at several time intervals (0, 6, 12, 24, and 48 h) is illustrated in Table 1. Compared to the negative control (PBS + OAs), which had 0% degradation and residual levels, the positive control (PBS + MAs) had 0% degradation and residual levels of 50 and 49.9 g/mL at 0 and 48 h, respectively. MA was degraded to varying degrees in all treatments. In this study, the mixed treatment using all organic acids demonstrated higher degradation efficiencies and lower residual MA levels (*p* < 0.05) than individual treatments at all concentrations and all-time intervals, reflecting the synergistic actions between organic acids in the degradation of MA in the mixed treatment. Accordingly, organic acid quadri-mix revealed the greatest MA degradation% (*p* < 0.05) of 95.9, 96.8, and 97.3% at concentrations of 1, 1.5, and 2%, respectively, after 48h, indicating that the combined treatment maintains unique advantages for in vitro MA degradation since organic acids are natural metabolic molecules that are biocompatible and antimicrobial. In degradation strategies, the quadri-mix may provide a high number of multiple functional groups for the combined acids. Subsequently, this may regulate the crosslinking of harmful bio-matrixes and dynamic binding locations for additional conjugation and degradation of molecules, including MA [2]. In biomaterial science, Su et al. [19] discovered that the addition of MA to citric-based polymers was crosslinked through free radical polymerization, thus enhancing the chemical structures, swelling ratio, and degradation rates of these polymers.

Concerning the individual treatments based on the type of organic acid, we detected that the MA degradation percentage for examined acids was 95.7% for citric acid, >95.6% for lactic acid, >94.4% for malic acid, and >94.1% for tartaric acid. Numerous natural and synthetic organic acids contain acidic and basic groups, which account for their physical, chemical, and biological properties. Moreover, an earlier study revealed that citric acid could exert maximum germicidal activity at low pH, mainly between 3.1 and 4.7 [20]. At low pH, uncharged and undissociated citric acid can flow through the mycobacterial membrane. Furthermore, citric acid can cause the deterioration of enzymes, proteins, DNA, extracellular membranes, and cell death [21]. Another underlying mechanism is that citric acid can increase cellular permeability by chelating ions in the cellular envelope; thus, it may inhibit the uptake of vital nutritional components by the cell envelope, resulting in cell death [22]. Conversely, Zhang et al. [23] found that malic acid did not affect the cell composition at a concentration higher than 1%, but it was relatively more effective at a concentration of more than 1.2%.

The current research determined that organic acid degradation ability against MA depends on the concentration of their ionized molecules in PBS buffer. Accordingly, citric acid (CA) is a triprotic acid (CAH3) with three carboxylic groups that can dissociate into three protons and then produce equilibriums of three definite negatively charged molecules (dibasic, CAH^2−^ and tribasic, CA^3−^) in the solution. The negatively charged CA molecules are toxic and thought to deactivate the external bacterial membrane components by destabilizing or sequestering vital metals from the medium or solution. In contrast, the lower degradation efficacy observed in MA treated with some organic acids is probably due to a relative increase in protective vis toxic impacts of monobasic vis di- and tribasic forms of these molecules, respectively, as previously described by Buchanan and Golden [24]. Furthermore, it is feasible that the efficiency of these acids could be influenced by the electrostatic differences between their molecules on the surface of MA and the mycobacterial membrane potential [25].

As hypothesized, MA degradation varied with organic acid concentration and the incubation time. Accordingly, all organic acids showed the highest MA degradation percentage (96 ± 2%) at concentrations of 2% up to 48 h. Furthermore, organic acids at 1 and 1.5% concentrations achieved a more desirable MA degradation percentage in the ranges of (92 ± 3%) at 1% and (94 ± 2%) at 1.5% of acids for 48 h. A similar investigation was conducted by Burel et al. [26], who found that 1% citric acid exhibited nonobvious cellular damage to *K. aerogenes* and *E. coli* cells, while the addition of 10% produced damaged cell walls with cavities and shrunken surfaces. Additionally, Fu et al. [27] observed that 99% of *S. aureus* and 96% of *E. coli* cells were destroyed by 14% of citric acid. Moreover, In et al. [28] found that *S. flexneri* and *S. dysenteriae* cells were inhibited at higher concentrations (2–5%) of citric and lactic acids than zero inhibition at 1%, with citric acid being a more potent antimicrobial than lactic acid. Our finding is inconsistent with El Baaboua et al. [7], who showed that tartaric acid, citric acid, and lactic acid prevented *S. typhimurium* at concentrations of 0.312, 0.625, and 0.156%, respectively.

Another underlying mechanism of MA degradation efficiency by organic acids was reported in a previous study [26], which demonstrated the superior efficacy of citric acid as an antimicrobial candidate at higher pH value was proposed based on zeta potential aspects, which reveal a more negatively charged microbial surface. This pH-dependent increase in surface charge may have rendered the cells potentially more sensitive towards chelating agents such as citric acid^3−^ (CA^3−^) that interact with membrane-stabilizing divalent metals. Thus, this increase in surface charge could enhance the bacterial dependency on the membrane-stabilizing divalent metal ions. Determining the impact of pH on organic acid efficacy should permit a more systematic means for selecting organic acids to optimize microbial pathogens’ inactivation and/or inhibition.

When the treatments were compared based on incubation time, the shortest interval (6 h) had the most insignificant effect on residual MA level (30–10 μg/mL) than other time intervals. Consistent with the findings of Buchanan and Golden [24], a minimum of numerous days (8 days) was necessary to realize bacterial destruction in the medium treated with organic acids. Similarly, In et al. [28] confirmed that by increasing the incubation time from 2 to 10 h, 40–100% of *S. flexneri* and *S. dysenteriae* were damaged at a concentration of 0.5% citric or lactic acid.

### 2.2. Mycolic Acid Degradation Efficiency by Fruit and LAB Extracts in PBS Buffer

Results listed in Table 2 reveal that MA degradation was affected by applying fruit and LAB extracts in a concentration-dependent manner at various time intervals. At all concentrations and incubation hours, the percentage of MA degradation for mixed extract (lemon+ apple+ grape+ cell-free supernatant of *L. acidophilus*) increased significantly (*p* < 0.05) at the highest level compared with controls and individual treatment. Accordingly, the MA degradation percentage reached its highest value at 2% of mixed extract and was 99.2% after 48 h. The mixed extract achieved a slightly lower MA degradation percentage with 97.16 and 98.10% after 48 h, at 1 and 1.5% concentrations, respectively. Gill et al. [29] illustrated that the combination of antimicrobial compounds together might cause different interactions with different impacts that may be synergistic, antagonistic, or additive. This finding explains the synergistic actions among examined extracts against MA degradation. The combination allowed for the assimilation of minor doses of each fruit extract and cell-free supernatant of *L. acidophilus*. According to Park et al. [30], the multicomponent mixture of *R. officinalis*, *C. sinensis*, citric acid, and polylysine suppressed *S. aureus*, *E. coli*, *B. cereus*, *S. enteritidis*, and *L. monocytogenes* with the complete destruction of all bacteria within 24 h of treatment.

Comparing the individual treatments, the MA degradation efficiency for lemon extract was higher (*p* < 0.05) than that for other extracts with a degradation percentage of lemon: 97.32 > *L. acidophilus* supernatant: 96.16 > grape: 95.48% > apple: 94.30% at a concentration of 2% after 48 h. We found that the shortest incubation time (6 h) had the minimum impact on residual MA level (29–60 μg/mL) compared with other incubation hours. The magnitude of MA degradation percent for all treatments at 1 and 1.5% of extracts was lower than that for the 2% concentration, ranging between 94 ± 3% and 95 ± 3% at 1 and 1.5% of acids for 48 h, respectively.

Previous studies have revealed that various lemon extracts potently inhibited a wide range of foodborne pathogens, such as *S. aureus*, *S. lutea, L. monocytogenes*, *E. coli*, *P. aeruginosa*, *S. typhimurium*, *Bacillus* spp., *Micrococcus* spp., *Aspergillus*, and *Penicillium* spp. [31,32]. Behera et al. [33] demonstrated that apple extract exerted a higher antimicrobial effect against *E. faecalis* and *S. mutans* than grape extract. Similarly, Malaviya and Mishra [34] reported that apple extracts were effective against *B. subtilis*, *S. aureus*, *E. coli*, and *P. aeruginosa* with inhibition levels of 2 and 10 mm. Additionally, Rhodes et al. [35] illustrated that grape juice and grape extracts had potent antimicrobial activity against *L. monocytogenes* but were ineffective against *B. cereus*, *E. coli*, and *S. aureus*. Furthermore, Kralik et al. [36] reported that cell-free supernatants of *Lb. plantarum* and *Lb. helveticus* reduced *Mycobacterium avium* subsp. *paratuberculosis* cells by >2 log^10^.

This finding agrees with Fahim et al. [18], who indicated that the antimicrobial activity of lemon balm extract against *M. smegmatis* and *M. bovis* had minimum inhibitory concentration rates of 12.5 and 50 μg/mL, respectively. In addition, Sandoval-Montemayor et al. [17] confirmed that the citral fraction of fruit peels (*C. aurantiifolia*) exhibited activity against *M. tuberculosis* H37Rv with a minimum inhibitory concentration value of 50 μg/mL. Indeed, fruits are one of the significant sources of antimicrobial agents. Consequently, considering the complex juice mixture for each fruit type is essential for producing several bioactive components and their metabolites, which may synergistically affect human health [37]. Therefore, in the present study, the mixture of fruit extracts displayed the maximum degradation efficiency against MA. Despite the data obtained on the antimicrobial ability of lemon, apple, grape, and cell-free supernatant of *L. acidophilus*, the combination of these extracts was first discussed here in our study.

### 2.3. Mycolic Acid Degradation Efficiency by Acid Quadri-Mix and Extract Quadri-Mix in Karish Cheese

Animal-based foods have been thought to be one of the main reasons for bovine TB in humans, particularly in developing countries such as Egypt. Thus, there is an urgent need to address this issue by preventing the proliferation of mycobacteria in milk and products with the use of safe and natural valuable products. Therefore, a degradation experiment of MA by organic acid quadric-mix (citric + lactic + malic + tartaric acid) was performed in the model Karish cheese to assess the MA degradation efficiency of the quadric-mix. Similarly, the MA degradation ability of the extract quadric mix (lemon + apple + grape + cell-free supernatant of *L. acidophilus*) was also evaluated. The experiment was conducted under varied mix concentrations (1, 1.5, and 2%) at five-time intervals (0, 6, 12, 24, and 48 h).

As depicted in Table 3, the overall results reflect a significant reduction (*p* < 0.05) in MA in Karish cheese compared with negative and positive controls, and both of them recorded 0% degradation. The residual MA in the positive control was consistent, with zero degradation percent for 48 h. In contrast, the MA degradation efficiency of the extract quadric mix achieved the best degradation percentage of 97.92, 98.54, and 99.50% at concentrations of 1, 1.5, and 2% at 48 h, respectively. Although the organic acid quadric-mix succeeded in degrading MA, the degradation ability was lower than (*p* < 0.05) that of the extract quadric-mix with 96.30, 97.28, and 98.04% percentages at concentrations of 1, 1.5, and 2%, respectively.

Regarding the effect of incubation time on MA degradation, we found that MA was primarily degraded by Karish cheese at the longest incubation time (48 h) for extract or organic acid quadric mix compared with other incubation hours and controls.

Due to the presence of certain elements that can change molecules’ polarity, most organic acids are highly soluble in organic solvents [38]. Accordingly, this study found that MA degradation values for organic acids and extracts were higher in Karish cheese than in PBS buffer, which could be attributed to the chemical composition of cheese, including fat, protein, and lactose, all of which can alter the property and activity of organic acids. Aside from the additional production of organic acids in raw milk and dairy products through the hydrolysis of milk fat, the addition of acidifiers (citric and lactic acids), microbial proliferation (lactic, formic, acetic, and pyruvic acids), and carbohydrate fermentation of LAB and regular bovine biochemical metabolism (citric acid) also play a role [39]. Furthermore, our study reveals that MA degradation values for extract quadri-mix were higher than those for acid quadri-mix in Karish cheese. This finding can be attributed to the type of fruit since significant amounts of different organic acids are found in lemon, apple, and grape, which can synergistically act to maximize their MA degradation efficiency. Another reason is that *L. acidophilus* can yield organic acids, such as lactic and citric acids, hydrogen peroxide, or diacetyl in the media (cheese), which have antimicrobial activities [36]. Therefore, this investigation was supported by further analysis of organic acid levels in the tested extracts using HPLC.

### 2.4. Organic Acid Portfolio of Individual and Mixed Extracts Determined by HPLC

**Figure 1:**
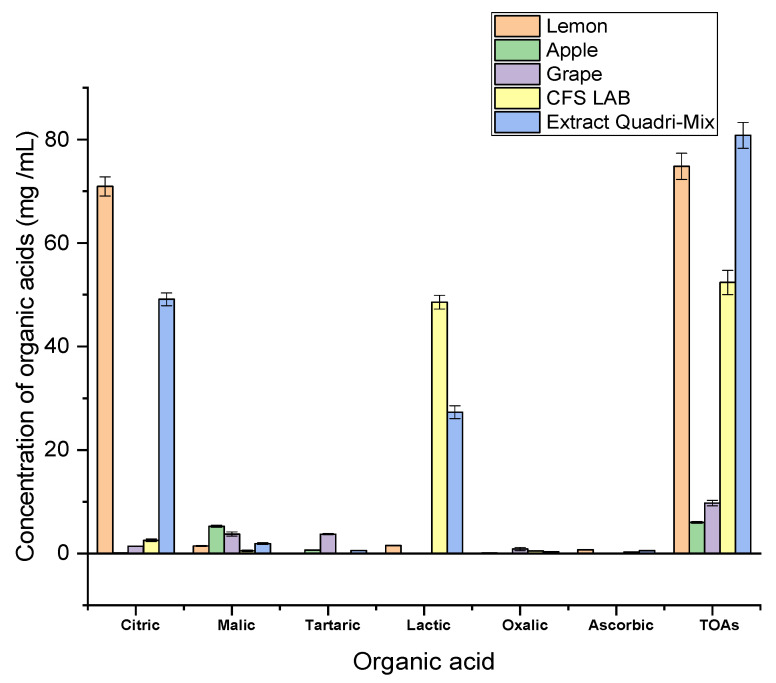
Concentrations of organic acids in lemon, apple, grape, cell-free supernatant of *L. acidophilus*, and quadri-mix extract (lemon + apple + grape + cell-free supernatant of *L. acidophilus*) determined by HPLC (mg/mL). TOAs (total organic acids), CFS LAB (cell-free supernatant of lactic acid bacteria).

The results of total and individual organic acids are demonstrated in Figure 1. The organic acids analyzed were citric, malic, tartaric, lactic, oxalic, and total organic acids. We noticed that the extract quadri-mix had the maximum organic acid content of 80.81 ± 2.48 mg/mL. The lemon extract had the highest (*p* < 0.05) total organic acids level of 74.82 ± 2.54 mg/mL. Citric acid was the most abundant in lemon and extracted quadri-mix with values of 70.94 ± 1.85 and 49.13 ± 1.23 mg/mL, while malic acid constituted the primary acid in apple and grape, and its values were 5.25 ± 0.21 and 3.75 ± 0.41 mg/mL, respectively. Although tartaric acid was identified in the lowest amount, it was a predominant acid in grape and apple, with 3.73 ± 0.14 and 0.65 ± 0.02 mg/mL values, respectively, but it was undetectable in the cell-free supernatant of *L. acidophilus*. Lactic acid was mainly found in the cell-free supernatant of *L. acidophilus* and extract quadri-mix with values of 48.57 ± 1.33 and 27.31 ± 1.24 mg/mL, respectively, while it was absent in apple and grape. Our findings are consistent with those obtained by Buyuktuncel [40], who found citric, malic, and ascorbic acid’s most abundant organic acids in citreous fruits.

Similarly, Zhang et al. [41] found the highest levels of tartaric and malic acids in grapes, with values of 55.72–60.07% and 28.54–39.52% of the entire organic acids, respectively. Furthermore, Yang et al. [42] confirmed that malic acid was apples’ most plentiful organic acid, with concentrations ranging from 2.8 to 6.9 mg/mL. In a study by Chen et al. [43], it was reported that lactic acid was the most predominant acid in cell-free supernatant of *Lactobacillus kefiri* among examined acids.

Assessment of organic acids in fruit extracts is crucial for their quality, acceptability, and process controls. The organic acids portfolio differs in fruits depending on their species, age, maturation, environment, light, temperature, and rainfall [44]. Additionally, it is necessary to note that fruits can link with the environment and other organisms. Consequently, their chemical structure and the level of active ingredients, including organic acids, can be very dissimilar [45]. Organic acids of microbial origin are considered postbiotics, exhibiting their wide-spectrum antimicrobial effect, synergistic activities with other metabolites, and great heat stability [46].

These findings explain that the maximum MA degradation efficiency of analyzed extracts was related to organic acid concentration, with the maximum degradation reported for extract quadri-mix followed by the lemon extract. Consequently, the intrinsic organic acids in extract quadri-mix enable it to degrade MA, suggesting that this extract is uniquely beneficial for application as a bio-preservative in the food industry and prospective medical applications where the antimicrobial potential is needed.

### 2.5. Sensorial Properties of Extract Quadri-Mix-Fortified Karish Cheese

In the current study, Karish cheese demonstrated grades (much-like > 8.0) for all sensory attributes before and after adding extract quadri-mix. When comparing the quadri-mix-extract-fortified cheese to the control, we discovered that odor and taste were the most important features in the sensory evaluation of Karish cheese. Except for odor and taste, the results in Table 4 reveal that all sensory scores are not significantly different (*p* > 0.05) between cheeses.

The mean odor and taste values were 8.7 and 8.9 for T2 (1.5 g of mixed extract in fortified cheese) and T3 (2.0 g of mixed extract in fortified cheese), respectively, which were higher than those for the control (8.5 and 8.4 for odor and taste respectively), while the minimum values reported for T1 (1.0 g of mixed extract in fortified cheese) were 8.4 and 8.2 for odor and taste, respectively.

Organic acids are essential for producing the ideal flavor, taste, aroma, and viscosity and improving the shelf-life of dairy products. They are also used as additives to stabilize or increase the palatability of dairy foods [47]. The desired odor and taste may be developed in fortified cheese due to the properties of some acids, such as malic acid, which has a flat and sour taste that remains in the mouth without imparting a burst of flavor [48]. Furthermore, tartaric acid has a powerful sour taste and a grape-like flavor [49]. These results are higher than those obtained by Tarse and Rick [50], who found that odorant taste scores for fresh Ethiopian cheese fortified with the citric acid solution were 6.0 and 5.0, respectively.

Color mean values for fortified cheeses (T2 and T3) vs. control were 8.8 vs. 8.7, respectively, while T2-fortified cheese had the lowest value of 8.6. The most predominant organic acids in the extract quadri-mix were citric acids, which are colorless and odorless, maximizing the color scoring of fortified cheese [51].

Excellent scores of both texture and appearance were reported for T3-fortified cheese to be 9.0 and 8.9, while their scores in T2-fortified cheese were 8.8 and 8.6, respectively. The overall acceptance rating showed the same value of 8.5 in T1- and T2-fortified cheeses, while the highest value (8.9) was reported for T3-fortified cheese compared with the control (8.6).

**Figure 2:**
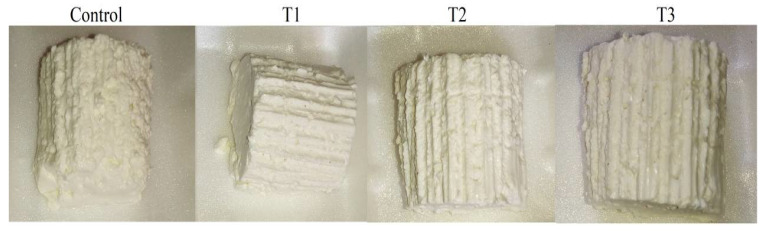
Fortified Karish cheeses: C: Control cheese (normal cheese without treatment) and T1, T2 and T3 are supplemented cheese (laboratory-made Karish cheese fortified by quadr-mix extract of degradants (lemon+ apple + grape+ cell free supernatant of *L. acidophilus*); T1 = Mix extract (1 g) (1 g extract quadri-mix/100 g Karish cheese); T2 = Mix extract (1.5 g) (1.5 g extract quadri-mix/100 g Karish cheese); T3 = Mix extract (2 g) (2 g extract quadri-mix/100 g Karish cheese)).

Organic acids can promote curd stability and character. Consequently, T2- and T3-fortified cheeses appeared smooth, creamy, moist, and soft with a rubbery and more homogeneous texture (Figure 2). This finding can be attributed to acidification reactions and low pH levels that cause weakness of the casein network and prevent casein hydration and solubility [52]. Similarly, Esmaeilzadeh et al. [53] reported a rubbery texture of Kurdish Kope cheese due to the presence of citric and lactic acids. The firmness and fragility of the T1-fortified cheese were minimal (Figure 2). It could be due to the inadequate concentration of the used extract (1%), which failed to achieve the maximum sensorial scores. Another reason is that increased moisture content diminishes the protein network, resulting in slightly firmer cheese. The moist and soft texture of cheese might be due to the high hygroscopic property of some organic acids, mainly citric acid, which is highly solubilized in water (62.07%), facilitating water retention and softness of cheese, as previously explained by Dalman [51].

In this study, the accepted sensory features for untreated cheese may be associated with the added salt, which can help to develop flavor, and make the curd firmer, as previously described by Smith and Hong-Shum [54]. The firmer appearance observed in the control cheese (Figure 2) could be attributed to its lower moisture content and compacted structure, as previously reported by Hassan et al. [55]. Furthermore, some organic acids, such as citric acid and lactic acid, are naturally present in raw milk, which can improve the sensory properties of produced cheese [56]. Since the organic acids used in this study are weak in nature, they can successfully lower the pH of milk from 6.6–6.8 of natural milk to 4.0–4.8, causing casein precipitation with excellent impacts on textural and appearance scores. When the pH of milk is decreased by adding organic acid, some milk components such as phosphate, citrate, and carboxylic acids form highly protonated molecules. These molecules neutralize the κ-casein part found on the surface of the casein micelle, causing the loss of solvency and electrostatic equilibrium and consequent clustering of casein micelles. Furthermore, the colloidal calcium phosphate, which is found inside the casein micelle, alters the micelle’s internal structure. All these factors contribute to casein precipitation [57].

Textural features might be related to acidification indices and pH, as previously confirmed by Robles-Rodriguez et al. [58]. Furthermore, the appearance scores of Karish cheese reported here were meaningfully affected by applying the extract quadri-mix.

Concerning the impact of acid type and concentration on the sensory features of precipitated casein, we noticed that all sensory scores were excellent for T2- and T3-fortified cheeses due to the application of mixed extract (lemon + apple + grape+ cell-free supernatant of *L. acidophilus*) with ideal concentrations of 1.5 and 2%. Acidification is one of the ancient practices in the food industry used to manufacture drinks, bread, cheese, and other foods, and it has no negative effect on the sensory features and nutritional value of organic acids [59]. Therefore, in our study, we used Karish cheese as an acid-coagulated model to assess the organic acid degradation efficiency and evaluate the fortified cheese’s sensory features without affecting organic acid activities.

## 3. Materials and Methods

### 3.1. Chemical and Reagents

All chemicals and reagents utilized in this study were purchased from Fluka and Sigma-Aldrich (Cairo, Egypt). Moreover, the used solvents, including methanol, acetone, chloroform, *n*-hexane, and acetonitrile, in addition to all culture media, were supplied by Merck (Darmstadt, Germany). MA from Mtb (bovine strain)—(Catalog Number M4537, CAS RN 37281-34-8) and organic acids, including citric acid, malic acid, tartaric acid, and lactic acid, were obtained from Sigma-Aldrich, Germany. The fruit materials of lemon, apple, and grape were bought from a fruit shop in Alexandria, Egypt. Probiotic cultures of *Lactobacillus acidophilus* were obtained from Microbiological Resources Centre (MIRCEN), Faculty of Agriculture, Ain Shams University, Cairo, Egypt.

### 3.2. Preparation of Standards

The standard solution of MA was prepared in *n*-hexane at a 10 mg/mL concentration and was diluted in 0.5 M PBS (pH 7.2) to the highest concentration of 50 µg/mL. Organic acids (citric acid, malic acid, tartaric acid, and lactic acid at a purity of 99–100%) were mixed with sterile distilled water to reach concentrations of 1.0, 1.5, and 2.0%. The fruit materials were peeled, sliced, blended, and filtered. The extracts were dried at 50 °C in Freeze Dryer (Telstar Model 50, Barcelona, Spain), and the powders were diluted with deionized water at concentrations of 1.0, 1.5, and 2.0 mg/mL [60].

### 3.3. Examination of Organic Acid Compounds in Fruit Extracts by HPLC

The mixed standard stock solution of organic acids containing 1000, 2000, 700, and 400 mg/L of citric acid, malic acid, tartaric acid, and lactic acid, respectively, was prepared in ultrapure water and stored at 4 °C. The HPLC analysis was carried out according to Violeta et al. [61], and a diode array detector (DAD) was utilized.

### 3.4. Probiotic Culture Cell-Free Supernatant Preparation

*Lactobacillus acidophilus* was propagated in de Man, Rogosa, and Sharpe (MRS) broth, according to Hamad et al. [62]. The supernatant was collected, filter-sterilized, then dried up using a vacuum freeze dryer (Model FDF 0350, Gyeonggido, Republic of Korea), and the collected powder was weighed.

### 3.5. Assessment of Mycolic Acid Degradation in PBS Buffer

MA was degraded using organic acids (citric acid, malic acid, tartaric acid, and lactic acid), fruit extracts (lemon, apple, and grape), and cell-free supernatant of (*Lactobacillus acidophilus*) in individual and mixed forms. In Eppendorf tubes, degradation effects were examined in PBS containing 50 µg/mL of MA. The assessment was conducted at different concentrations of degradants (1, 1.5, and 2%) and at various time intervals (0, 6, 12, 24, and 48 h). The samples were incubated with a continual shaking rate of 40 cycles/min at 37 °C for 30 min. They were then centrifuged at 25 °C and 3000 rpm/5 min, and 1 mL of the supernatant was drawn for HPLC analysis. The result was compared with the positive control (50 μg of MA/mL) and negative control (degradants suspended in PBS). The Degradation percentage was calculated using the following equation:

$$\mathrm{The}\;\mathrm{Degradation}\;\mathrm{percentage}=\frac{1-\mathrm{MA}\;\mathrm{concentrations}\;\mathrm{in}\;\mathrm{the}\;\mathrm{existence}\;\mathrm{of}\;\mathrm{degradant}}{\mathrm{MA}\;\mathrm{concentrations}\;\mathrm{in}\;\mathrm{the}\;\mathrm{standard}\;\mathrm{sample}}\times 100$$

### 3.6. Determination of Mycolic Acid Degradation Using HPLC

#### 3.6.1. Mycolic Acid Derivatization

The mycobacteria also used a purified MA extract, and the sample was derivatized after the purification. MA derivatization was conducted according to the procedure of Brugnera et al. [63]. The catalyst dicyclohexyl-18-crown-6 ether (0.005 mol/L) and the derivatization reagent bromophenacyl bromide (0.1 mol/L) were firstly suspended in acetonitrile reagent. The MA was derivatized to bromophenacyl bromide esters by adding 20 mg of KHCO_3_, 1 mL of chloroform, and 50 µL of derivatization reagent. Further, the tubes were heated at 105 °C/20 min. Samples were then cooled and filtrated by a 0.45 µm pore-sized filter.

#### 3.6.2. Chromatographic Analysis

An Agilent 1200 HPLC (Agilent Technologies, Palo Alto, CA, USA) equipped with a Waters XBridge C18 column (2.1 × 150 mm, 3.5 μm) and photodiode array detector was set for MA separation using a gradient of 100% of solvent A (99% methanol, 1% 5 mM ammonium acetate) to 100% solvent B (79% *n*-propanol, 20% *n*-hexane, 1% 5 mM ammonium acetate) at a temperature of 45 °C. The flow rate was 0.32 mL/min over a 45 min run period. The ESI/APCI-MS analysis was carried out according to Sartain et al. [64]. The calibration curve was performed using different concentrations of MA. The limit of detection (LOD) level of MA was 7.60 ng/mL. The limit of quantification (LOQ) was 20.01 ng/mL. On the other hand, the linear range for the MA is 0.5 to 100 ng/mL.

### 3.7. Evaluation of Degradation Effect of Acid Quadri-Mix and Quadri-Mix Extract in Karish Cheese

Karish cheese was made from 4 kg of bovine milk divided into five 0.5 kg parts. Negative control (cheese only), positive control (cheese with MA 50 µg/g), organic acid-treated cheese (cheese with mixed organic acid 1, 1.5, 2% + MA 50 µg/g), and extract (fruit and LAB)-treated cheese (cheese with mixed extract (fruit and LAB) 1, 1.5, 2% + MA 50 µg/g). Milk was analyzed for MA detection and heated at 63 °C for 30 min, then kept at 32–35 °C, in which MA treatments were applied, and CaCl_2_ (0.9 g) was added. Subsequently, milk was kept in earthenware pots at room temperature until the formation of curd. The cream layer was skimmed, and curd was ladled into the cheese mat, pressed, and hung until complete syneresis, then cut into small pieces and stored in the refrigerator. Samples were collected to determine residual MA and degradation percentage and compared with controls [65].

### 3.8. Sensory Evaluation of Extract Quadri-Mix-Fortified Karish Cheese

Sensory evaluation of control and treated cheese was conducted as described by Wood et al. [66] with some modifications. The samples were examined at room temperature using a 10-point Hedonic scale and a semi-trained panel consisting of 20 members familiar with the consumption of cheese samples.

### 3.9. Statistical Analysis

Data were expressed as mean values of duplicates± standard deviation at the statistical significance level of *p* < 0.05 and then evaluated via the one-way analysis of variance (ANOVA) using the Tukey’s test and IBM SPSS statistics 23 software program, ABM, Armonk, New York, NY, USA.

## 4. Conclusions

In PBS and Karish cheese, fruit extract and probiotic *L. acidophilus* quadri-mix had higher degradation efficiency (*p* < 0.05) against MA than organic acid quadri-mix, individual acids, and sole extracts, especially at 1.5 and 2% concentrations. Through the acidification phenomenon, fruit extract combined with *L. acidophilus* quadri-mix was shown to degrade MA and prevent its accumulation in casein during the cheese-making process. Citric acid was more effective than other acids at degrading MA. In the sensory evaluation, a relevant application may include the innovative and eco-friendly fortified Karish cheese, prepared with extract quadri-mix and had the most desirable sensorial attributes. This study could be further extended by including other cheeses and organic acids and testing MA degradation under different conditions, such as concentration, temperature, humidity, storage, and ripening procedures. In addition, it is strongly suggested that future studies include bacterial culture tests to determine whether MA degradation will also influence bacterial survival in food sources.

## Figures and Tables

**Table 1:** Mycolic acid degradation efficiency by organic acids in PBS buffer.

Matrix	OA (%)	MA (µg/mL)	Residual MAs (µg/mL)					Degradation%
			0 h	6 h	12 h	24 h	48 h	
PBS + OAs (−Ve control)	1.00	0	0	0	0	0	0	0
	1.50	0	0	0	0	0	0	0
	2.00	0	0	0	0	0	0	0
PBS + MAs (+Ve control)	0.00	50.00 ± 0.26 ^a^	50.00 ± 0.66 ^a^	50.00 ± 0.32 ^a^	50.00 ± 0.70 ^a^	49.99 ± 0.26 ^a^	49.99 ± 0.15 ^a^	0
Citric acid +PBS + MAs	1.00	50.00 ± 0.47 ^a^	50.00 ± 0.16 ^a^	25.0 ± 0.34 ^de^	13.5 ± 0.96 ^c^	7.25 ± 0.47 ^c^	4.26 ± 7 ^c^	91.5 ± 0.87 ^cd^
	1.50			20.0 ± 0.48 ^g^	10.7 ± 0.44 ^ij^	6.12 ± 036 ^cd^	3.17 ± 0.60 ^n^	93.6 ± 0.59 ^c^
	2.00			15.0 ± 0.70 ^h^	8.23 ± 0.23 ^j^	5.13 ± 0.31 ^e^	2.14 ± 0.19 ^ef^	95.7 ± 0.47 ^ab^
Malic acid + PBS + MAs	1.00			30.0 ± 0.18 ^b^	16.3 ± 0.80 ^b^	9.12 ± 0.78 ^b^	5.17 ± 0.06 ^b^	89.7 ± 0.17 ^d^
	1.50			27.0 ± 0.29 ^c^	13.5 ± 0.60 ^c^	6.92 ± 0.11 ^cd^	3.45 ± 0.60 ^d^	93.1 ± 0.50 ^c^
	2.00			24.0 ± 0.95 ^de^	12.6 ± 0.60 ^d^	5.94 ± 0.71 ^d^	2.81 ± 0.60 ^e^	94.4 ± 0.36 ^b^
Tartaric acid + PBS + MAs	1.00			29.0 ± 0.66 ^b^	14.3 ± 0.67 ^bc^	7.53 ± 0.82 ^c^	5.32 ± 0.47 ^b^	89.4 ± 0.74 ^d^
	1.50			26.0 ± 0.32 ^d^	13.0 ± 0.50 ^d^	5.71 ± 0.81 ^d^	3.52 ± 0.50 ^d^	93.0 ± 0.60 ^c^
	2.00			23.0 ± 0.69 ^f^	11.8 ± 0.87 ^d^	4.89 ± 0.12 ^l^	2.95 ± 0.61 ^cd^	94.1 ± 0.74 ^b^
Lactic acid + PBS + MAs	1.00			28.0 ± 0.87 ^bc^	13.1 ± 0.42 ^c^	6.63 ± 0.69 ^cd^	3.15 ± 0.80 ^d^	93.7 ± 0.12 ^c^
	1.50			25.0 ± 0.70 ^de^	11.6 ± 0.36 ^e^	4.74 ± 0.73 ^e^	2.53 ± 0.17 ^e^	94.9 ± 0.58 ^b^
	2.00			20.0 ± 0.43 ^g^	9.13 ± 0.83 ^f^	3.92 ± 0.60 ^f^	2.21 ± 0.39 ^ef^	95.6 ± 0.80 ^b^
OA quadric mix + PBS + MAs	1.00			20.0 ± 0.60 ^g^	9.53 ± 0.20 ^f^	4.23 ± 036 ^e^	2.06 ± 0.28 ^f^	95.98 ± 0.53 ^ab^
	1.50			15.0 ± 0.74 ^h^	6.76 ± 0.60 ^g^	3.16 ± 0.48 ^fg^	1.61 ± 0.1 0 ^g^	96.8 ± 0.44 ^a^
	2.00			10.0 ± 0.42 ^i^	4.81 ± 0.36 ^h^	2.32 ± 0.40 ^g^	1.35 ± 0.64 ^h^	97.3 ± 0.60 ^a^

PBS: Phosphate buffered saline; MAs: Mycolic acid standard; OAs, Organic acids; −Ve (negative control without mycolic acid standard); +Ve (positive control with mycolic acid standard). Means with different superscripts in small letters on the same column are significantly different (*p* < 0.05).

**Table 2:** Mycolic acid degradation efficiency by fruit extracts and cell-free supernatant of *L. acidophilus* in PBS buffer.

Matrix	OA (%)	MA (µg/mL)	Residual MAs (µg/mL)					Degradation%
			0 h	6 h	12 h	24 h	48 h	
PBS + FEs (−Ve control)	1.00	0	0	0	0	0	0	0
	1.50	0	0	0	0	0	0	0
	2.00	0	0	0	0	0	0	0
PBS + MAs (+Ve control)	0.00	50.0 ± 0.07 ^a^	50.0 ± 0.32 ^a^	50.0 ± 0.37 ^a^	50.0 ± 0.47 ^a^	50.0 ± 0.69 ^a^	50.0 ± 0.38 ^a^	0
Lemon + PBS + MAs	1.00	50.0 ± 0.55 ^a^	50.0 ± 0.67 ^a^	18.0 ± 0.22 ^e^	10.3 ± 0.02 ^d^	4.96 ± 0.71 ^cd^	2.52 ± 0.71 ^d^	95.0 ± 0.72 ^bc^
	1.50			14.0 ± 0.22 ^f^	7.14 ± 0.67 ^e^	3.16 ± 0.56 ^i^	1.96 ± 0.64 ^e^	96.1 ± 0.60 ^b^
	2.00			9.00 ± 0.51 ^e^	4.36 ± 0.77 ^h^	2.24 ± 0.13 ^j^	1.34 ± 0.12 ^f^	97.3 ± 0.17 ^ab^
Apple + PBS + MAs	1.00			29.0 ± 0.66 ^b^	14.8 ± 0.39 ^b^	7.53 ± 0.34 ^b^	4.13 ± 0.21 ^b^	91.7 ± 0.07 ^e^
	1.50			26.0 ± 0.28 ^c^	12.6 ± 0.27 ^c^	6.51 ± 0.12 ^b^	3.67 ± 0.63 ^bc^	92.7 ± 0.22 ^d^
	2.00			22.0 ± 0.37 ^d^	10.7 ± 0.11 ^d^	5.46 ± 0.82 ^c^	2.85 ± 0.19 ^d^	94.3 ± 0.20 ^bc^
Grape + PBS + MAs	1.00			27.0 ± 0.23 ^c^	14.3 ± 0.22 ^b^	6.93 ± 0.63 ^b^	3.87 ± 0.02 ^bc^	92.36 ± 0.30 ^d^
	1.50			24.0 ± 0.802 ^d^	11.6 ± 67 ^c^	5.95 ± 0.33 ^c^	2.78 ± 0.27 ^d^	94.4 ± 0.37 ^bc^
	2.00			20.0 ± 0.22 ^e^	9.83 ± 0.73 ^d^	4.76 ± 0.17 ^d^	2.26 ± 0.37 ^de^	95.5 ± 0.61 ^b^
CFS of (*L. acidophilus*) + PBS + MAs	1.00			23.0 ± 0.22 ^d^	11.1 ± 0. 23 ^cd^	6.05 ± 0.57 ^c^	3.19 ± 0.41 ^c^	93.6 ± 0.22 ^c^
	1.50			18.0 ± 0.73 ^e^	9.26 ± 0.63 ^d^	4.13 ± 0.13 ^d^	2.27 ± 0.18 ^de^	95.5 ± 0.27 ^b^
	2.00			14.0 ± 0.62 ^f^	6.75 ± 0.82 ^e^	3.36 ± 0.52 ^f^	1.92 ± 0.37 ^e^	96.2 ± 0.92 ^b^
Extract quadri mix + PBS + MAs	1.00			15.0 ± 0.22 ^f^	6.92 ± 0.46 ^e^	3.61 ± 0.40 ^e^	1.42 ± 0.41 ^f^	97.2 ± 0.20 ^ab^
	1.50			10.0 ± 0.74 ^g^	5.13 ± 0.76 ^f^	2.23 ± 0.71 ^g^	0.95 ± 0.55 ^g^	98.1 ± 0.47 ^a^
	2.00			6.00 ± 0.12 ^h^	3.16 ± 0.82 ^i^	1.71 ± 0.63 ^h^	0.41 ± 0.22 ^h^	99.2 ± 0.32 ^a^

PBS: Phosphate buffered saline; Mas: Mycolic acid standard; CFS: Cell-free supernatant; FEs: Fruit extracts; OAs, Organic acids; −Ve (negative control without mycolic acid standard); +Ve (positive control with mycolic acid standard). Means with different superscripts in small letters on the same column are significantly different (*p* < 0.05).

**Table 3:** Mycolic acid degradation efficiency by acid quadri-mix and extract quadri-mix in Karish cheese.

Matrix	OA (%)	MA (µg/mL)	Residual MAs (µg/mL)					Degradation%
			0 h	6 h	12 h	24 h	48 h	
Acid quadri-mix (citric, malic, tartaric, and lactic) + Karish cheese (-Ve control)	0	0	0	0	0	0	0	0
Extract quadri-mix (lemon, apple, grape, and *L. acidophilus*) + Karish cheese (−Ve control)	0	0	0	0	0	0	0	0
Karish cheese + MAs (+Ve control)	0	50.0 ± 0.22 ^a^	50.0 ± 0.51 ^a^	49.9 ± 0.02 ^a^	49.0 ± 0.12 ^a^	48.5 ± 0.41 ^a^	48.0 ± 0.74 ^a^	0
Acid quadri-mix + Karish cheese + MAs	1.00	50.00 ± 0.91 ^a^	50.00 ± 0.71 ^a^	19.00 ± 0.66 ^b^	9.03 ± 0.63 ^b^	3.92 ± 0.57 ^b^	1.85 ± 0.12 ^b^	96.30 ± 0.84 ^c^
	1.50			14.00 ± 0.32 ^c^	7.21 ± 0.54 ^c^	3.04 ± 0.31 ^bc^	1.36 ± 0.36 ^b^	97.28 ± 0.02 ^b^
	2.00			9.00 ± 0.41 ^d^	4.87 ± 0.73 ^e^	2.53 ± 0.87 ^c^	0.98 ± 0.81 ^c^	98.04 ± 0.22 ^ab^
Extract quadri-mix + Karish cheese + MAs	1.00			13.00 ± 0.25 ^c^	5.13 ± 0.91 ^d^	2.36 ± 0.12 ^c^	1.04 ± 0.47 ^bc^	97.92 ± 0.17 ^b^
	1.50			9.00 ± 0.71 ^d^	7.75 ± 0.32 ^c^	4.92 ± 0.42 ^b^	0.73 ± 0.15 ^c^	98.54 ± 0.78 ^a^
	2.00			4.00 ± 0.80 ^e^	2.18 ± 0.45 ^f^	1.05 ± 0.36 ^d^	0.25 ± 0.62 ^d^	99.50 ± 0.54 ^a^

MAs: Mycolic acid standard; Acid quadri-mix = citric+ malic + tartaric+ lactic; Extract quadri-mix = lemon + apple + grape + cell free supernatant of *L. acidophilus*; −Ve (negative control without mycolic acid standard); +Ve (positive control with mycolic acid standard). Means with different superscripts in small letters on the same column are significantly different (*p* < 0.05).

**Table 4:** Sensorial scoring of extract quadri-mix-fortified Karish cheese.

Treatment/Group	Sensorial Scores Mean ± (SD)					
	Color	Odor	Taste	Texture	Appearance	Overall Acceptance
Control	8.7 ± 0.64 ^a^	8.5 ± 0.82 ^a^	8.4 ± 0.80 ^a^	8.6 ± 0.67 ^a^	8.7 ± 0.64 ^a^	8.6 ± 0.64 ^a^
T1 Mix extract (1 g) (1g extract quadri-mix/100 g Karish cheese)	8.6 ± 0.80 ^a^	8.4 ± 0.82 ^a^	8.2 ± 0.90 ^a^	8.6 ± 0.66 ^a^	8.3 ± 1.28 ^a^	8.5 ± 0.77 ^a^
T2 Mix extract (1.5 g) (1.5 g extract quadri-mix/100 g Karish cheese)	8.8 ± 0.40 ^a^	8.7 ± 0.60 ^a^	8.7 ± 0.64 ^a^	8.8 ± 0.40 ^a^	8.6 ± 0.80 ^a^	8.5 ± 0.78 ^a^
T3 Mix extract (2 g) (2 g extract quadri-mix/100 g Karish cheese)	8.8 ± 0.60 ^a^	8.9 ± 0.30 ^a^	8.9 ± 0.30 ^a^	9.0 ± 0.00 ^a^	8.9 ± 0.31 ^a^	8.8 ± 0.20 ^a^

Extract quadri-mix = lemon+ apple + grape+ cell-free supernatant of *L. acidophilus*. Sensory scoring: extremely excellent = 9, excellent = 8, very, very good = 7, very good = 6 good = 5, fair = 4, poor = 3, very poor = 2, extremely bad = 1. Means with different superscripts in small letters on the same column are significantly different (*p* < 0.05).

## Data Availability

The data presented in this study are available on request from the corresponding author.
